# Supplementary material for: Factors associated with registration for organ donation among clinical nurses
Source: PLoS One. 2021 Feb 19;16(2):e0247424. doi: 10.1371/journal.pone.0247424 (PMC7895373; doi:10.1371/journal.pone.0247424)
Supplement: S1 File — (DOCX) [file pone.0247424.s001.docx]

**Knowledge of organ donation (15 items, answer on yes, no, and unknown)**

1. Post-circulating death indicates the donors were cardia arrest and death

2. The deceased organs were recovered from brain death donors or post-circulating death donors

3. To become a potential post-circulating death donor, s/he needs to have an end-stage disease, receive hospice care, and should be determined by two physicians for the eligibility.

4. The post-circulating death was confirmed 5 minutes after cardiac arrest

5. Providing any medication was prohibited after the confirmation of post-circulating death and removing the life support machine

6. Insertion of femoral arterial tubing for infusion and anti-coagulation medication was allowed for maintaining the utilization of organs after circulating death

7. There is no age limit to be a brain death organ donor

8. The brain death procedure needs to be checked twice, with an interval of 12 hours

9. Every physician was allowed to do the brain death evaluation regardless of their specialty

10. In the case of accidental death, the organ donation becomes a possibility when obtaining approval from the prosecutor’s consent

11.The time of death of the organ donor can be determined at the end of the donation operation

12. Donors who died of brain death, not due to disease, must notify the prosecutor in the jurisdiction after the first brain death evaluation

13. After the donor’s organs are recovered, the appearance of the remains will be properly restored

14. The government has the discretion to subsidize the funeral expenses for the relatives of the donor after death

15. According to the regulations on human organ transplantation, health professionals should take the initiative to ask patients or family members about their willingness of organ donation

**Attitude toward organ donation (13 items, Likert scale 1-5)**

1. I will consider to be a donor if my organs are still functional

2. Donating one’s organs after death are moral and can help other people

3. The organs after death are not useful, so they can be donated

4. Organ donation could help people in need

5. Organ donation is a manifestation of human love

6. Organ donation could help people rebirth, which is a wonderful thing

7. The human body is just a temporary shell

8. After the organ donation, the person will live in another form of life

9. It is immoral to harvest one's organs

10.Organ donation could cause distress and sorrow for family members

11. It is important to "keep the whole body" after death

12. Donating organs will make the deceased donor feel not peaceful

13. Organ donation makes the body incomplete that will affect the afterlife negatively

**Cultural myth (5 items, Likert Scale 1-5)**

1.Discussing deceased organ donation brings bad luck

2. In folk belief, it is important for the body to remain intact after death

3. In folk belief, people retain the concept of going home in one breath (to die at home)

4. Fear of organ recovered procedure after death

5. The folks have the notion that " Parents give us the body, and dare not to hurt, it is the filial piety "

**Practical difficulties involved in organ donation (9 items, Likert scale 1-5)**

1. The current notification mechanism inside and outside the hospital cannot detect potential organ donors immediately

2. It is difficult to collaborate with other departments in relation to organ procurement and donation

3. Delayed brain death evaluation is due to the inability of relevant hospital units’ cooperation

4. It is difficult to cooperate with the time or process of organ donation

5. Staffs do not understand the legal process of organ donation

6. Family members can't wait for the brain death evaluation (the time takes too long)

7. Staffing is a big issue that can make organ donation difficult

8. The organ recruitment team is not proactive in organ recruitment

9. The organ donation process is cumbersome

**Convenience of registration (3 items, Likert scale 1-5)**

1. The registration process of organ donation is simple and clear

2. The donated organs are allocated and used properly

3. The way or method to register the organ donation was convenient

**Feasibility of the opt-out principle (1 item, Likert scale 1-5)**

I think that the “opt-out” principle could implement in our country to promote organ donation

**The third-grade priority policy (1 item, Likert scale 1-5)**

I think that the third-grade priority has the potential to improve the organ donation rate
