# Supplementary material for: Factors associated with registration for organ donation among clinical nurses
Source: PLoS One. 2021 Feb 19;16(2):e0247424. doi: 10.1371/journal.pone.0247424 (PMC7895373; doi:10.1371/journal.pone.0247424)
Supplement: S2 File — (PDF) [file pone.0247424.s002.pdf]

Knowledge of organ donation (15 items, answer on yes, no, and unknown)

1.心臟死是指器官捐贈者已心跳停止且死亡
2.大愛器官來源是由醫師判定為腦死或心臟死的病人所捐贈的
3.必須經由 2 位專科醫師判定為末期病患且已同意安寧醫療才可以進行心臟死器官捐贈
4.根據規定，心臟死後器官捐贈必須在病人心跳停止 5 分鐘後，方可確定死亡
5.心臟死後器官捐贈者在同意器官捐贈並撤除維生系統後，就不可以使用任何藥物
6.為維持透過心臟死後器官捐贈所捐出器官的可用性，可置放股動脈導管以灌入冷卻輸液和注射抗凝血劑
7.經判定腦死之器官捐贈者沒有絕對年齡限制
8.腦死判定程序需進行 2 次，每次間隔約需 12 小時
9.任何科別屬性的醫師都可以執行腦死判定
10.腦死判定後若為意外死亡，須由檢察官判定後開立檢察官同意書才可以捐贈器官
11.器官捐贈者的死亡時間為捐贈手術結束時間
12.非因疾病死亡的腦死捐贈者在第一次腦死判定後須通知轄區內的檢察官
13.捐贈者的器官取出後，遺體外觀會妥善恢復
14.政府得對死後捐贈者之親屬，酌予補助喪葬費
15.依據人體器官移植條例，醫護人員應主動詢問病人或家屬的器捐意願

Attitude toward organ donation (13 items, Likert scale 1-5)

1.如果我的器官還有功能可以捐贈，我會考慮
2.器官捐贈是積德與助人的事
3.死後的器官是用不到的，所以可以捐出來
4.器官捐贈可以幫助有需要的人
5.器官捐贈是人類大愛的展現
6.器官捐贈可以助人重生，是一件美好的事
7.人的身體只是暫時的軀殼
8.器官捐贈後，我會以另外一個型態活著
9.摘取一個人的器官是不道德的事
10.器官捐贈會造成家人的苦惱與哀傷
11.死亡後「保持全屍」是很重要的
12.器官捐贈摘取器官，會讓往生的捐贈者身心靈不安寧
13.器官捐贈後，身體不完整對來世會造成影響

Cultural myth (5 items, Likert Scale 1-5)

1.簽器官捐贈同意書會觸霉頭
2.民間觀念需保留全屍
3.民間保留一口氣回家(死在家中)的觀念
4.對死後器官被摘除感到恐懼

5.民間有「身體髮膚受之父母，不敢毀傷，孝之始也」的觀念

Practical difficulties involved in organ donation (9 items, Likert scale 1-5)

1.醫院內外的現行通報機制無法即時發覺潛在器官捐贈者

2.醫院相關單位無法配合器官勸募及器捐作業

3.因醫院相關單位無法配合而延遲腦死判定作業

4.無法配合器官捐贈的時間或流程

5.不瞭解器官捐贈的法律流程

6.家屬無法/不耐等候腦死判定

7.醫護人員之夜班及人力問題，無法配合器官捐贈流程

8.器官勸募團隊對器官勸募不積極

9.器官捐贈處理流程繁瑣

Convenience of registration (3 items, Likert scale 1-5)

1.器官捐贈登記流程簡潔明確

2.捐贈的器官能被妥善分配與運用

3.簽署器官捐贈卡的管道方便

Feasibility of the opt-out principle (1 item, Likert scale 1-5)

就我對我們國家民情的理解，有關國外所謂的「表態反對」立法應已可在台灣推行

The third-grade priority policy (1 item, Likert scale 1-5)

相信三親等優先順序可以促進器官捐贈率
