# Supplementary material for: Factors associated with registration for organ donation among clinical nurses
Source: PLoS One. 2021 Feb 19;16(2):e0247424. doi: 10.1371/journal.pone.0247424 (PMC7895373; doi:10.1371/journal.pone.0247424)
Supplement: Attachment — Submitted filename: response and revision.docx [file pone.0247424.s003.docx]

Dear editor and reviewers:

Thank you very much for your recommendation. We have revised the abstract, method, results, discussion, and other sections according to your comments. Please let us know whether there is anything else that is needed.

Sincerely,

Authors

PONE-D-20-30483
Factors associated with the registration of organ donation among clinical nurses

1. For more information on PLOS ONE's expectations for statistical reporting, please see https://journals.plos.org/plosone/s/submission-guidelines.#loc-statistical-reporting. Please update your Methods and Results sections accordingly.

Response: We have updated the statistical reporting in the methods and results section. In Data analysis section, “Descriptive statistics were used to estimate central tendency (mean) and spread (standard deviation) for continuous data such as age and years of clinical experience, while frequencies and percentages were used for categorical data including gender, educational level, and marital status. The chi-square test was used to examine the associations between categorical variables and registration status (registered or unregistered). The independent samples t-test was used to examine the differences in continuous variables between registered and unregistered participants. To analyze important factors in the context of organ donation registration, first, univariate logistic regression was conducted to analyze the effect of variables that showed significant differences in the t-test and chi-square test. Variables that were statistically significant in the univariate logistic regression (p < 0.05) were then included together in the multivariate logistic regression to examine their associations with organ donation registration.”

2.Please include additional information regarding the survey or questionnaire used in the study and ensure that you have provided sufficient details that others could replicate the analyses..

Response: We have attached the questionnaires.

3.Thank you for stating the following financial disclosure:

 [The funders had no role in study design, data collection and analysis, decision to publish, or preparation of the manuscript]. Please include your amended statements within your cover letter; we will change the online submission form on your behalf.

Response: We have included the statement within the cover letter.

**Review Comments to the Author**

Reviewer #1:

1.Please prepare a structured abstract (purpose, materials and methods, results, conclusion and keywords). Also add some demographic information in the results section such as mean age and % of male or female.

Response and revision: Thank you for the recommendation. We have revised the abstract as “Purpose: Healthcare professionals play an important role in the organ donation. The aims of this study was to examine the registration rate of organ donation and its related factors among clinical nurses.

Material and methods: This cross-sectional and correlational study using mailed questionnaires to collected data from four geographical areas and three hospital levels in Taiwan from June 6 2018 to August 31, 2018. Two thousand and thirty-three clinical nurses participated in this study.

Results: Participants’ mean age was 34.47 years, and 95.7% were women. Of them, 78.3% were willing to donate their organs and 20.6% had registered for organ donation after death. The results of logistic regression showed that in the personal domain, older in age (OR (odds ratio) = 1.03, p < 0.001), better knowledge of organ donation (OR = 1.09, p < 0.001), and a positive attitude toward organ donation (OR = 2.91, p < 0.001) were positive factors associated with organ donation registration, while cultural myths (OR = 0.69, p < 0.001) were negatively correlated. In the policy domain, convenience of registration procedure (OR = 1.45, p < 0.001) was positive factor associated with registration. A gap between the willingness to donation and real registered was happened among clinical nurses.

Conclusions: The personal domain related factors were important factors related to the registration of organ donation. Improving knowledge and inculcate positive cultural beliefs about organ donation among clinical nurses was recommend. Hospital managers also suggested cooperate with government policies to provide appropriate in-service training, policy incentives, and establish an efficient registration process.

Key words: attitude, culture, knowledge, nurse, organ donation, policymaking”

2.Please update your bibliography citation in accordance with guideline of journal.

Response and revision: Thank you for your recommendation. We have revised the citation.

3. Page 4, lines 106-108, it is unclear and need revision.

Response and revision: Thank you for your recommendation, we have revised as “The authors reviewed the published literature and the details available on the TORSC website; this information, together with their personal experiences, was used to identify the factors associated with deceased organ donation registration. These factors were categorized into three domains: personal, healthcare setting, and policy. The content of the questionnaires is presented in S1 and S2.”

4. Please attach your questionnaire as a supplementary file. In my opinion, your well-designed questionnaire can help authors from other countries to conduct similar research in this field and promote the literature.

Response and revision: Thank you for the recommendation. We have attached the questionnaire as supplementary file.

5. Please clear your sampling method.

Response and revision: Thank you for your recommendation, in sample section, we have added “Participants were recruited through simple stratified sampling based on geographic area and hospital level.”

6.The mentioned response rate is not in accordance with the literature but the reason may be giving gifts to participants.

Response: in data collection section we have mentioned that “In order to increase the response rate, each questionnaire included a gift certificate worth US $3.5 as a token of gratitude.”

7.Page 9, results section, paragraph one is lengthy. Please delete the subject that mentioned in table 1.

Response and revision: Thank you for your recommendation. We have shortened the paragraph one as “The demographic characteristics of the sample were comparable to those of employed nurses in Taiwan. Of the 2033 participants, 1592 (78.3%) were willing to donate their organs after death, and 419 (20.6%) had registered for organ donation on their National Health Insurance cards. There were significant differences in age, years of clinical experience, and religion between the groups. The registered group had higher knowledge of organ donation, believed in fewer cultural myths, and had a more positive attitude toward organ donation than the unregistered group.”

8. Please add a paragraph about the strength, and weakness or limitation of the study in discussion section.

Response and revision: Thank you for your recommendation. We have added a paragraph in discussion section “A strength of this study is its large sample size, allowing the results to be representative of clinical nurses from different regions and hospital levels. The characteristics of the sample are also consistent with those of practicing nurses in Taiwan. However, a weakness of this study is that the influence of healthcare setting factors could not be confirmed. Given its important role in promoting organ donation, the effects of factors related to the healthcare setting on registration need further investigation. In addition, while the reliability and validity of the questionnaire, which was developed for the purpose of this study, were confirmed, they require further examination.”

Reviewer #2: 

1- Very large sample size increases the power of the study so much that even a subtle effect, rather than a practical effect, can become significant. It is the case in this study with a sample size equal to 2033, the very trivial effect of the factors such as age (OR=1.03) and knowledge of organ donation (OR=1.09) become statistically significant without having a practical interpretation. Therefore, the authors should consider the effect of the large sample size in the interpretation of the results.

Response and revision: Thank you for your recommendation, in discussion section, paragraph three, line257-259, we have revised as “. It should be noted that this may simply have been a result of the large sample size, since the OR was small. The association of age with organ donation registration may need more investigation” and line 262-263, “Although the coefficient of knowledge of organ donation was small, knowledge is the basis of practice.”

2- Table 1 shows a significant relationship between religion and registration of organ donation. As the authors stated in the discussion, the non-significant effect of the religion in the logistic regression model could be as a result of multicollinearity with the cultural myths. The crude regression must be performed on each variable separately before entering the variables into the multiple regression. This way, the effect of each variable can be studied separately and it becomes clear whether the variable does not affect the outcome or its nonsignificant effect is due to collinearity with other variables in the study.

Response and revision: Thank you for your recommendation, we have performed the crude regression for each variable. In the results section, line 220-222, “Univariate logistic regression was conducted for each variable that was significantly different between the registered and unregistered groups. The results are presented in Table 4.”
