# Supplementary material for: Factors associated with registration for organ donation among clinical nurses
Source: PLoS One. 2021 Feb 19;16(2):e0247424. doi: 10.1371/journal.pone.0247424 (PMC7895373; doi:10.1371/journal.pone.0247424)
Supplement: Attachment — Submitted filename: response and revision20201230.docx [file pone.0247424.s004.docx]

**Response and revision**

**Comment from the editor**

*I will condition my final acceptance recommendation to a full proof reading of the manuscript to ensure that the English flows better than it does now.*

**Response:** The author appreciate the opportunity to edit our manuscript to improve its flow. Our responses to your comment are below.

**Abstract**

**(page 2, line 24-43)**

Purpose: Healthcare professionals play an important role in the organ donation process. The aim of this study was to examine the organ donation registration rate and related factors among clinical nurses.

Material and methods: In this cross-sectional, correlational study, we used mailed questionnaires to collect data from four geographical areas and three hospital levels in Taiwan from June 6 to August 31, 2018. Two thousand and thirty-three clinical nurses participated in this study.

Results: Participants’ mean age was 34.47 years, and 95.7% were women. Of them, 78.3% were willing to donate their organs and 20.6% had registered for organ donation after death. The results of logistic regression showed that in the personal domain, higher age (odds ratio (OR) = 1.03, p < 0.001), better knowledge of organ donation (OR = 1.09, p < 0.001), and a positive attitude toward organ donation (OR = 2.91, p < 0.001) were positively associated with organ donation registration, while cultural myths (OR = 0.69, p < 0.001) were negatively correlated. In the policy domain, the convenience of the registration procedure (OR = 1.45, p < 0.001) was positively associated with registration. A gap between willingness to donate and actual registration was observed.

Conclusions: Personal factors played an important role in organ donation registration. Therefore, efforts to improve knowledge and inculcate positive cultural beliefs about organ donation among clinical nurses are recommended. There is also a need to cooperate with government policies to provide appropriate in-service training and policy incentives and establish an efficient registration process.

**Introduction**

**(page 3, line57)**

However, the donation rate still has potential for improvement

**(page3, Line 66-67**)

At a time of shock, this puts families in a difficult position, but being aware of the patient’s wishes because of their registration status can facilitate decision-making

**(page4, line 70-71**)

Therefore, promoting registration for organ donation is considered an important task for countries that adopt the opt-in principle.

**(page 4, line 89-90)**

Therefore, the aims of this study, focused on clinical nurses, were to investigate the registration rate for organ donation and examine associated factors in this population.

**Sample and setting**

**(page5, line 93-100)**

This study employed a cross-sectional, correlational design. The inclusion criterion was clinical registered nurses employed in hospitals, regardless of their years of experience, gender, or specialty. Participants were recruited through simple stratified sampling based on geographic area and hospital level. As per the governmental definition, hospitals were categorized as the following: tertiary medical center, regional hospital, and local hospital level. The final 265 hospitals recruited for the sample had the following geographic distribution—north: 106, middle: 77, south: 66, and east: 16. By hospital level, they were divided as follows: 19 tertiary medical centers, 83 regional hospitals, and 163 local hospitals.

**Study variables and measurement tool**

**(page5, line 103-105)**

Participants were asked to answer two yes/no questions: one concerning their willingness toward organ donation and the second concerning whether they had registered for deceased organ donation on the National Health Insurance card.

**Ethical consideration**

**(page 7, Line 159-162)**

The cover letter included the study purpose, procedure, and details concerning participants’ personal information, such as assuring them of anonymity, confidentiality, and that the published results would contain only de-identified data. Participation was voluntary.

**Data collection**

**(page 8, line 164-171)**

Data collection was via the mailed questionnaires. After the institutional review board approved the research proposal, TORSC staff helped us contact hospital administrators and seek their assistance with distributing the questionnaire to nurses. Then, the official research description, an explanatory cover letter, and the questionnaire were mailed to the hospital administration department. Based on the nursing capacity of each institution, 100–150 questionnaires were mailed to medical centers, 20–50 to regional hospitals, and 5–10 to local hospitals. In order to increase the response rate, each questionnaire included a gift certificate worth US $3.5 as a token of gratitude. After completion, nurses were asked to return the questionnaires by mail in pre-stamped envelopes.

**Results (page 12, line 220-221)**

Multivariate logistic regression (forced entry model) was used to examine the factors associated with registered or unregistered status.

**Discussion**

**(page 13, line 237-240)**

Nurses who were older, had greater knowledge of organ donation, held more positive attitudes toward organ donation, did not believe very strongly in cultural myths, and perceived the registration procedure as convenient were most likely to register for organ donation.

**(Page 13, line 245-247)**

However, in the present study, the figure was lower than a previous study that reported a registration rate of 47% [18]. The difference could be a result of variations in cultural backgrounds and organ donation policies across countries [2].

**(page 13-14, line 248-253)**

The association of age with deceased organ donation registration varies. In the general population, older people, that is, those aged 50 years and above, might not consider registering for deceased organ donation because of poor health [18, 20]. However, in this study, older nurses were inclined to register for deceased organ donation. Older clinical nurses have more experience in clinical care than younger nurses, which may influence their attitude and behavior concerning organ donation [10].

**(page 14, line 264-267)**

Generally, cultural myths are negatively associated with organ donation registration. While in this sample there did not appear to be a dominant cultural myth, beliefs such as talking about organ donation registration bringing bad luck, respect for the corpse and the need to keep it intact, and fear about the organ recovery procedure were mentioned.

**(page 14, line 267-274)**

Fear about the surgical recovery procedure may be rooted in misunderstandings about deceased organ donation [13–15, 17]. The belief that one’s body should remain intact after death is common in Asian cultures [22, 23]. Further, shielding one’s parents’ bodies from harm is considered an expression of filial piety [24, 25]. Donating vital organs is considered to result in one’s inability to “survive” in the world after death [13, 24]. Some Buddhists believe that the soul needs at least 24 hours to integrate and prepare to leave a deceased body, and that harm done to the body after physical death can still be “felt” in the soul; therefore, during this time, the body must not be tampered with [26, 27].

**(page 14-15, line 275-276)**

When a person is alive, it is taboo to talk about the possibility of their subsequently becoming a deceased organ donor as it makes people uncomfortable.

**(page15-16, line 298-299)**

Notably, higher deceased organ donation rates have been observed in countries that implement the opt-out rather than the opt-in principle [28]
